# Emergency endovascular treatment of cavernous internal carotid artery acute bleeding with flow diverter stent: a single-center experience

**DOI:** 10.1007/s00701-020-04517-0

**Published:** 2020-08-18

**Authors:** Andrea Giorgianni, Edoardo Agosti, Alberto Terrana, Fabio Pozzi, Giorgio Sileo, Luca Nativo, Sergio Balbi, Alessandro Motta, Paolo Castelnuovo, Davide Locatelli, Mario Turri-Zanoni

**Affiliations:** 1 Department of Neuroradiology, ASST Sette Laghi, Varese, Italy; 2 Division of Neurosurgery, Department of Biotechnology and Life Sciences, University of Insubria, Via Guicciardini, 9, 21100 Varese, Italy; 3 Division of Otorhinolaryngology, Department of Biotechnology and Life Sciences, University of Insubria, Varese, Italy; 4 Department of Anesthesiology e Resuscitation, ASST Sette Laghi, Varese, Italy; 5 Head and Neck Surgery & Forensic Dissection Research Center (HNS&FDRc), Department of Biotechnology and Life Sciences, University of Insubria, Varese, Italy

**Keywords:** Cavernous carotid artery, Acute vascular injury, Flow diverter stent, Skull base surgery, Endoscopic endonasal, Hadad flap

## Abstract

**Background and objective:**

To describe our single-center experience in the treatment of cavernous internal carotid artery (ICA) acute bleeding with flow diverter stent (FDS), as a single endovascular procedure or combined with an endoscopic endonasal approach.

**Methods:**

We analyze a case series of 5 patients with cavernous ICA acute bleeding, i.e., 3 iatrogenic, 1 post-traumatic, and 1 erosive neoplastic. After an immediate nasal packing to temporarily bleeding control, patients underwent digital subtraction angiography (DSA) to identify the site of the ICA injury. A concomitant balloon occlusion test (BOT) was performed, to exclude post-occlusive ischemic neurological damage. An FDS was placed with parallel intravenous infusion of abciximab in 3 cases and tirofiban in 2 cases. In two patients, an innovative “sandwich technique” combining the endovascular reconstruction with an extracranial intrasphenoidal cavernous ICA resurfacing with autologous flaps or grafts by endoscopic endonasal approach was performed.

**Results:**

No patient had periprocedural ischemic-hemorrhagic complications. All patients had a regular clinical evolution, without general complications or new onset of focal neurological deficits. No further bleeding occurred in 3 patients, while 2 cases experienced a mild rebleeding in a period ranging from 5 to 15 days after the endovascular procedure. In these two cases, we proceeded with an endoscopic endonasal procedure to resurface the exposed ICA wall in the sphenoid sinus.

**Conclusions:**

Although the treatment of choice for cavernous ICA acute bleeding remains the occlusion of the injured vessel, in cases of poor hemodynamic compensation at the BTO, the endovascular FDS emergency placement can be effective. A combined endoscopic endonasal technique to support the extracranial side of the vessel using autologous flaps or grafts can be performed to prevent the risk of rebleeding.

## Introduction

The internal carotid artery (ICA) acute bleeding is one of the most serious neurovascular emergencies that require rapid diagnostic framing and therapeutic targeting. Among the causes of ICA acute injury are traumatic damage, iatrogenic procedure, and neoplastic invasion. Among the carotid segments of the most difficult management in the case of acute rupture is the cavernous tract. In particular, in the case of cavernous ICA acute injury, the control of bleeding can be obtained using an endovascular procedure, endonasal endoscopic approach, or combined techniques [1–6].

Endovascular treatment can be based on either closing the injured vessel or repairing the vascular wall tear. To date, the occlusive technique is preferred to the reconstructive, given its best efficacy in bleeding control, both in the short and long term. However, the final choice between these two endovascular procedures depends on whether or not the sacrifice of the injured vessel can be tolerated by the patient in terms of possible post-occlusive neurological deficits. The hemodynamic compensation capacity can be estimated through the determination of the venous phase prominent delay of the injured vessel during balloon occlusion test (BOT) [7]: the greater the delay, the lower the hemodynamic compensation, the major the risk of post-occlusive ischemic damage of the territory downstream of the damaged vessel [8].

Different types of techniques and stents for the management of a cavernous ICA acute injury have been described in the literature. However, a study that offers a comprehensive evaluation of the emergency management of cavernous ICA acute bleeding with flow diverter stent (FDS) endovascular placement is lacking. The aim of this study is to describe our single-center experience in the management and treatment of acute cavernous ICA bleeding with FDS endovascular emergency placement, as an isolated procedure or a combined technique with an endoscopic endonasal trans-sphenoidal cavernous ICA wall reconstruction, providing indications, contraindications, and outcomes.

## Materials and methods

### Data collection

A retrospective review of neuroradiology registry data of patients treated with endovascular FDS placement from 2015 to 2019 was performed. Inclusion criteria were patients with cavernous ICA acute bleeding, with pathological BOT positive for post-occlusive ischemic risk, and treated in an emergency with FDS release. Of these, only patients over the age of 18 and who gave their informed consent were included.

Clinical, pathological, and radiological data were retrieved from the review of medical records and available radiological imaging and prospectively collected in a dedicated database. Clinical data included sex and age of the patient, cavernous ICA injury cause, treatment, complications, and outcome. Radiological data comprehended type of FDS and follow-up radiological investigations, while pathological data included the site and type of the lesion.

### Endovascular procedure

Regardless of cavernous ICA injury etiopathology, each patient underwent a digital subtraction angiography (DSA) to define the location and extension of the vascular wall injury. During the angiographic study, a BOT was performed to calculate the venous phase delay and assess the hemodynamic compensation capacity of the cerebral circulation [7]. Vascular anatomy was also studied, to select the FDS that potentially best suited the individual patient’s conformation.

In detail, in all 5 cases, DSA was performed in an emergency regimen, in general anesthesia with a femoral artery access. The BOT was immediately practiced and the blood pressure was maintained in a variable range between 70 and 80 mmHg for diastolic blood pressure and between 110 and 120 mmHg for systolic blood pressure. The parent vessel occlusion test was standardized to a duration of 10 min.

After a multidisciplinary discussion of each case, FDS was placed. During stent deployment, antiplatelet protocol included 8 mg of abciximab (used in 3 cases) or 10 mg of tirofiban (used in 2 cases). A subsequent 12-h infusion of a saline solution with the same antiplatelet drug (9 mg in 200 ml of saline solution at 17 ml/h for abciximab and 10 mg in 100 of saline solution at 11 ml/h for tirofiban) was performed.

Double antiplatelet therapy with clopidogrel 75 mg plus acetylsalicylic acid (ASA) 100 mg was initiated the day after the procedure and it was maintained for 1 month, while single antiplatelet ASA 100 mg therapy was maintained for other 3 months, for a total of 4 months of antiplatelet therapy.

### Endoscopic endonasal procedure

**Fig. 1:**
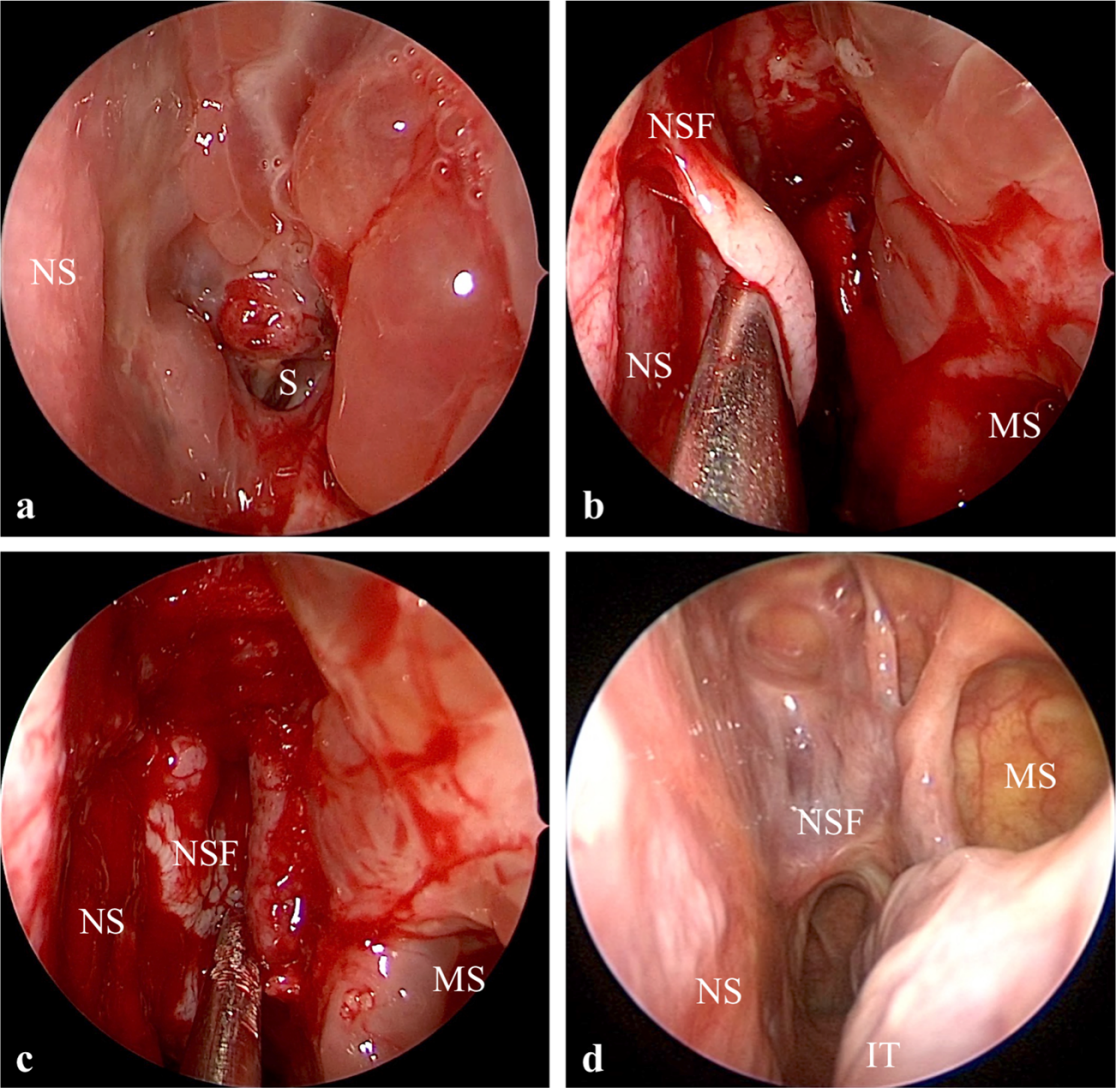
Endoscopic endonasal “sandwich technique” with Hadad flap harvesting (case #3). **a** Left sphenoidotomy. **b** Harvesting of left nasoseptal flap (Hadad flap). **c** Nasoseptal flap placed to cover the left sphenoidotomy. **d** Endonasal endoscopic image at 22 months, showing complete healing of the nasoseptal flap in the sphenoid as well as the nasal septum donor site

In two out of 5 cases of rebleeding after endovascular FDS emergency placement, an endoscopic endonasal trans-sphenoidal procedure was performed to resurface with autologous tissue the cavernous ICA injured wall. After a total spheno-ethmoidectomy, the lateral wall of the sphenoid sinus was inspected with angled scopes (Karl Storz, Tüttlingen, Germany) and the cavernous segment of the ICA was identified. The bony edges around the carotid pseudoaneurysm (PSA) were exposed and denuded from mucosa. The cavernous ICA wall was then covered with an autologous graft (i.e., inferior turbinal mucoperichondrium graft) in one case, and in the other patient, a pedicled nasoseptal Hadad flap was harvested and rotated to resurface the lateral wall of the sphenoid, including the cavernous tract of the ICA (Fig. 1a, b, c). According to this technique, a double reconstruction of the injured ICA wall was performed: an endovascular cavernous ICA endoluminal reconstruction by FDS positioning and an extracranial endonasal reconstruction with autologous flap or graft. We defined this innovative combined endovascular-endonasal procedure as “sandwich technique.” Nasal packing was placed and then removed after 48 h. A peri-operative third-generation cephalosporin was administered intravenously for 7 days.

### Follow-up

**Fig. 2:**
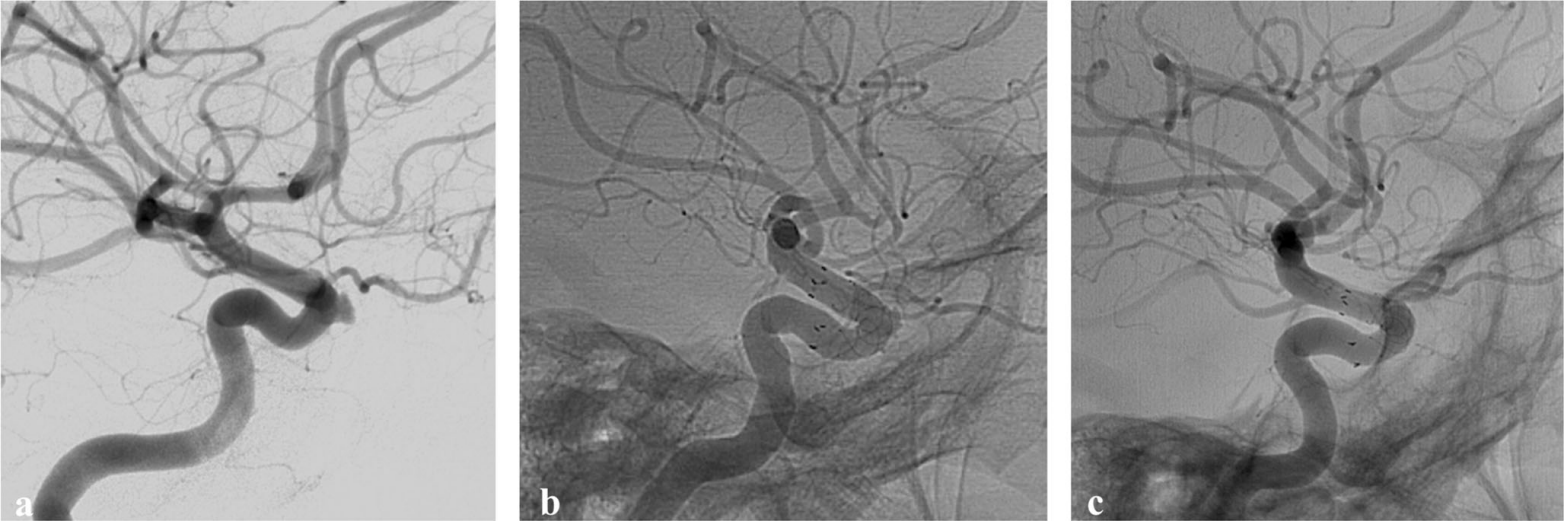
Case #3 is a 48-year-old woman admitted to our institution for sudden epistaxis. She was affected by recurrent chronic rhinosinusitis with nasal polyps treated at another center with radical spheno-ethmoidectomy with maxillary and frontal sinusotomies about 1 month before. A diagnostic CTA and DSA documented a PSA of the anterior profile of the left carotid siphon. An FDS (FRED, 4 × 12/18 mm) was placed. **a** Pre-procedural DSA showing PSA of the anterior genu right cavernous ICA segment. **b** Intra-procedural DSA showing FDS correct placement. **c** DSA at 3 months, demonstrating the regular cavernous ICA profile, without evidence of PSA recurrence

**Fig. 3:**
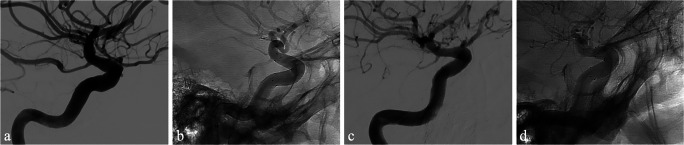
Case #4 is a 48-year-old man with pituitary GH-secreting macroadenoma. The lesion was approached with an endoscopic trans-sphenoidal paraseptal binostril approach. An intraoperative cavernous ICA medial wall damage occurred during the sphenoidotomy. Immediate bleeding control was achieved by direct packing with hemostatic agent (oxidized regenerated cellulose, Surgicel® Original, Ethicon, Inc., NJ, USA) of the sphenoid sinus and nasal cavity. The patient was immediately moved to the angiographic room to manage the injured vessel. **a** Pre-procedural DSA showing a PSA of the anterior genu right cavernous ICA segment. **b** Intra-procedural DSA showing FDS correct placement. **c** and **d** DSA at 12 months demonstrating the appropriate occlusion of the PSA (**c**) and correct FDS placement (**d**)

**Fig. 4:**
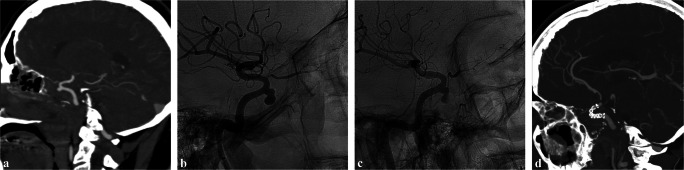
Case #5 was a 54-year-old male affected by poorly differentiated sphenoid sinus squamous cell carcinoma (pT4bN0M0), extended bilaterally to cavernous sinuses and with a 360° encasement of right ICA, previously submitted to chemotherapy (2 cycles of cisplatin and paclitaxel) and intensity-modulated proton beam therapy (70 Gy). The patient experienced massive epistaxis, investigated with diagnostic imaging, including CTA and DSA, which showed cavernous ICA PSA. The bleeding was controlled by FDS placement. **a** and **b** Pre-procedural CTA (**a**) and DSA (**b**) showing anterior genu right ICA cavernous segment PSA. **c** Intra-procedural DSA showing FDS correct placement. **d** CTA at 1 month demonstrating PSA resolution

The neuroradiological follow-up was carried out by CTA and DSA, following a standard scheme for each patient. After the FDS release, a DSA control was performed, to verify the FDS correct positioning, normal patency and flow of both ICA, and the reduced supply of contrast to the pseudoaneurysmatic collection from the vessel lumen. One month after the procedure, a CTA was performed in all patients. A control DSA was performed at 3 and 12 months after the procedure. Figures 2, 3, and 4 show the most representative images of the pre-procedural DSA, the positioning of the FDS, and the post-procedural controls with CTA at 1 month and DSA at 3 and 12 months in some of the treated cases.

When an endoscopic endonasal surgical procedure was performed to reconstruct the extracranial side of the ICA wall (“sandwich technique”), the patients were also followed with seriated endoscopic endonasal medications to maintain the patency of the sphenoid ostium and improve the mucosal healing of the surgical cavity, which usually can be obtained within 2 months after surgery (Fig. 1d).

## Results

We selected 5 patients treated in our center with emergency positioning of FDS on the ICA cavernous tract. Clinical, pathological, and radiological data are summarized in Table 1. Four of the 5 patients were male; the mean age was 47.4 years (range, 21–66 years).

**Table 1:** Clinical, radiological, procedural, and follow-up data of the five cases treated

	Sex	Age	ICA injury etiology	Appearance	Flow diverter	Periprocedural anticoagulant therapy	endoscopic endonasal Reconstruction	1-, 3-, 12-month follow-up	Status	Follow-up months
Case 1	M	21	Traumatic injury	Post-traumatic investigations	P64, 4 × 18 mm	Abciximab	No	CTA, DSA, DSA	Alive	65
Case 2	M	66	Pituitary adenoma	Intraoperative bleeding	PED, 4 × 20 mm	Abciximab	No	CTA, DSA, DSA	Alive	57
Case 3	F	48	Pansinusal polyposis in chronic rhinosinusitis	Epistaxis	FRED, 4 × 12/18 mm	Abciximab	Hadad nasoseptal flap	CTA, DSA, DSA	Alive	31
Case 4	M	48	Pituitary adenoma	Intraoperative bleeding	DED, 6 × 20 mm	Tirofiban	Inferior turbinal mucoperichondrium autologous graft	CTA, DSA, DSA	Alive	12
Case 5	M	54	Poorly differentiated squamous cell carcinoma of the sphenoid sinus	Epistaxis	FRED, 4 × 17/23 mm	Tirofiban	No	CTA, DSA, -	Exitus	18

*Abbreviations*: *CTA*, CT angiography; *DED*, Derivo embolization device; *DSA*, digital subtraction angiography; *F*, female; *FRED*, flow-redirection endoluminal device; *M*, male; *PED*, pipeline embolization device

During the DSA, the presence of a PSA of the cavernous ICA was evident in all cases. A carotid BOT [7] was performed during angiographic procedure, resulting in a prominent delay in venous phase of the ipsilateral hemisphere to the occluded ICA in all 5 patients. Furthermore, DSA highlighted the intrinsically curved anatomy of the cavernous ICA at the level of the carotid-ophthalmic passage, making the placement of a covered stent challenging. The endovascular emergency positioning of the FDS obtained a repair of the cavernous ICA tear, with bleeding control and initial slowing of the contrast diffusion in the PSA, in all cases. There was no immediate post-procedural rebleeding in any patient. No thrombo-embolic cerebral ischemic complications occurred after positioning the stent in any patient.

In two cases, a mild and temporary ICA rebleeding occurred in a period ranging from 5 (case #4) to 15 days (case #3), requiring further treatments to strengthen the reconstruction. In such cases, a combined endoscopic endonasal procedure (“sandwich technique”) was performed to resurface the outer side of the cavernous ICA, as previously described.

After a mean follow-up of 36.6 months (range, 12–65 months), 4 out 5 patients were alive without evidence of symptoms, without any tardive FDS-related complications, with patency of the intracranial circle at the last control. The remaining patient (case #5) died due to the intracranial progression of sinonasal cancer.

## Discussion

In this study, we showed our single-center preliminary experience in the management of cavernous ICA acute bleeding, describing for the first time a case series of patients treated in the emergency with endovascular FDS placement, as an isolated procedure or combined with an endoscopic endonasal trans-sphenoidal approach, demonstrating the absence of post-procedural ischemic-hemorrhagic complications in the short and long term.

Managing a cavernous ICA acute injury is a complex issue, as it involves rapid blood loss originating from a challenging to reach anatomical area. There are three options to treat such a condition: ICA sacrifice, ICA lesion embolization, and ICA endoluminal reconstruction [1–6, 9].

The ICA sacrifice is the treatment of choice and generally performed after proper verification of the hemodynamic compensation by vascular occlusion test (e.g., BOT). A variable low rate of permanent neurological ischemic deficits after ICA occlusion remains, on average estimated around 0.4% [7].

In the case of vascular wall lesions with high flow blood loss, the embolization technique represents the best solution, especially when the cavernous ICA acute injury is localized on the lateral wall of the vessel, towards the cavernous sinus. In this case, the blood collects forming a pseudoaneurysmatic pouch, until it breaks generating a carotid-cavernous fistula (CCF), with a direct flow from the carotid artery (higher pressure area) to the cavernous sinus (lower pressure area). The high-speed flow makes CCF treatment by flow diversion challenging given the unfavorable hemodynamics, although some cases of CCF effectively treated with endovascular FDS positioning have been reported in the literature [10–12].

Conversely, when the breach is located on the medial wall of the cavernous ICA, blood collects between the vessel wall and the lateral wall of the sphenoid sinus, forming a PSA. In some cases, the collection increases until the pressure of the PSA is such as to damage the thin bone of the sphenoid sinus lateral wall and pour it into the sphenoid cavity. In the case of PSA, the blood flow between the vessel and the pseudoaneurysmatic collection is limited and the FDS can effectively derive the flow and interrupt the discontinuity of the vascular wall.

Over the years, vascular endoluminal reconstruction with stents has emerged as a feasible alternative to occlusion of the damaged vessel. Several cases have been described in the literature where uncovered and covered polytetrafluoroethylene (PTFE) stents were placed to repair arterial tears. Celil et al. [2] and Chen et al. [13] report cases of cavernous ICA PSA treated effectively with covered stent implantation. Ruiz-Juretschke et al. [5] use an overlapping self-expanding uncovered stent for the treatment of post-traumatic cavernous ICA PSA, and Ko et al. [4] describe another case of PSA treatment with a balloon-expandable covered stent. However, these types of stents may have some disadvantages and limitations. The main problem is represented by the reduced flexibility, which limits its applicability to vessels with a tortuous course such as the cavernous and paraclival tract of the ICA. Besides, complications such as stent thrombosis and stent embolism, vascular dissection, malapposition, and endoleak can occur [6, 14–17].

A valid alternative to the uncovered and PTFE stents for the endovascular reconstruction of cavernous ICA tear is represented by the FDS. At present, there are few cases described in the literature of a cavernous ICA acute injury successfully managed with an FDS release. Some cases of iatrogenic cavernous ICA PSA treated in the late post-operative period with FDS are reported in the literature [1, 3, 12, 18]. Giorgianni et al. report two cases describing the emergency positioning of FDS for cavernous ICA acute injury, respectively, in a traumatic [3] and iatrogenic cavernous ICA tear [18]. Also Nariai et al. [19] report on a single patient who underwent successful treatment using an FDS for an iatrogenic intracranial internal carotid artery pseudoaneurysm following a cavernous ICA injury after endoscopic pituitary tumor resection via the trans-sphenoidal approach.

Recently, the use of coated FDS for the endovascular treatment of acute ruptured intracranial aneurysms has been described. Among the coated FDS proposed, hydrophilic polymer coating (HPC) [20, 21] and pipeline embolization device with shield technology (PED-shield) are currently available [19, 22, 23]. The advantage of coated FDS would be the post-procedural single antiplatelet therapy (SAPT), compared with the double antiplatelet therapy (DAPT) required in non-coated FDS. Early data collected on coated FDS in animal studies and small case series showed less thrombogenicity, faster endothelial growth, and comparable neointimal volume than other non-coated devices [19–23]. However, these are preliminary evidence, with the need for further investigation with a larger number of patients and a formal treatment registry [22]. It must be considered that in our case series, there were no hemorrhagic complications associated with the post-procedural DAPT. Therefore, there is still no demonstrated evidence of the need to use a SAPT in patients with FDS. The use of coated-FDS, as HPC and PED-shield, can instead certainly be useful for patients with hemorrhagic risk factors in whom DAPT is strongly discouraged (e.g., polytraumatized, thrombocytopenic patient).

Furthermore, we showed that the endoscopic endonasal resurfacing of the extracranial wall of the vessel using flaps or grafts can play a synergistic role with FDS endovascular treatment in repairing the vascular wall tear. This combined “sandwich technique” can offer a double support at intra- and extracranial level for the reconstruction of the cavernous ICA wall, reducing the risk of rebleeding. According to our experience, we propose the sandwich technique not as a standard of care in case of FDS placement but as a specific technique to manage rebleeding after FDS placement. This technique has not been yet described in the literature and the outcomes obtained in our two preliminary cases are encouraging, supporting the usefulness of this combined procedure.

It must be noted that FDS placement has some disadvantages, including the need of post-procedural double antiplatelet therapy, which should be maintained for a few months, and the long time necessary for a complete repair of the vessel [2, 18].

The main limitation of this study is represented by a small sample of patients analyzed. Further studies with a larger number of cases and involving multiple centers would be required to validate the results obtained in this study.

## Conclusions

In this study, we have shown the relevant role of the FDS endovascular emergency placement, which can be considered a safe and effective option, alternative to the definitive vascular occlusion, for selected cases of cavernous ICA acute bleeding, regardless of vascular injury caused. The actual indications of this technique are injuries placed in the medial wall of the cavernous ICA, in patients at high risk for neurological sequelae related to the ICA sacrifice, estimated on pre-operative radiological assessments. The combined endoscopic endonasal trans-sphenoidal resurface of the extracranial intrasphenoidal side of the cavernous ICA, which has been described in the present study for the first time, should be considered in selected cases of rebleeding to further strengthen the vessel wall reconstruction.

## Abbreviations

ASA: acetylsalicylic acid
BOT: balloon occlusion test
CT: computed tomography
CTA: CT angiography
DAPT: double antiplatelet therapy
DED: Derivo embolization device
DSA: digital subtraction angiography
F: female
FDS: flow diverter stent
FRED: flow-redirection endoluminal device
GCS: Glasgow coma scale
IMPT: intensity-modulated proton therapy
IT: inferior turbinate
iv: intravenous
M: male
MRI: magnetic resonance imaging
MS: maxillary sinus
NS: nasal septum
NSF: nasoseptal flap
PED: pipeline embolization device
PSA: pseudoaneurysm
PTFE: PolyTetraFluoroEthylene
S: left sphenoidotomy
SAPT: single antiplatelet therapy
